# Identification and characterization of two closely related virga-like viruses latently infecting rubber trees (*Hevea brasiliensis*)

**DOI:** 10.3389/fmicb.2023.1286369

**Published:** 2023-12-14

**Authors:** Ruibai Zhao, Xiaoqi Su, Fengjuan Yu, Zhu Liu, Xi Huang

**Affiliations:** ^1^College of Tropical Crops, Sanya Nanfan Research Institute of Hainan University, Sanya, China; ^2^School of Life Sciences, Hainan University, Haikou, China

**Keywords:** virus discovery, high-throughput sequencing, latent virus, rubber tree latent virus 1, rubber tree latent virus 2

## Abstract

A novel virga-like virus, provisionally named Rubber tree latent virus 2 (RTLV2), was identified from rubber tree (*Hevea brasiliensis*). It is a close relative of the previously reported Rubber tree latent virus 1 (RTLV1). The complete genomes of RTLV1 and RTLV2 were sequenced and comparatively analyzed in terms of genome organization, putative gene products and phylogenetic relationship. Both RTLV1 and RTLV2 have positive-sense single-stranded RNA genomes that encode seven open reading frames (ORFs), forming a similar genomic layout. In phylogenetic analyses based on replicase and coat protein amino acid sequences, RTLV1 and RTLV2 were clustered with unclassified virga-like viruses. They are distinct from currently recognized plant virus families. RTLV1 and RTLV2 can be distinguished from members of *Virgaviridae* by the presence of a putative coat protein duplex and a poly(A) tail at the 3′-terminus. The authenticity of RTLV1 and RTLV2 as infectious viruses was confirmed through field investigations and transmissibility assays. In conclusion, RTLV1 and RTLV2 represent a novel plant virus group that does not readily fit into current virus families.

## Introduction

The Para rubber tree (*Hevea brasiliensis* Muell.), a perennial deciduous woody plant in the family *Euphorbiaceae*, originates from the Brazilian Amazon. Today, it is widely cultivated in tropical areas worldwide, particularly in the Southeast Asia, for the commercial production of natural rubber (*cis*-1, 4-polyisoprene) (Liu et al., 2020). Rubber trees are the predominant source of natural rubber, comprising approximately one-third of the latex, which is essentially cytoplasm of the laticifers in the phloem (Tang et al., 2016). Commercial rubber tree plantations have been facing economic losses caused by several diseases, including the South American leaf blight (SALB) caused by *Pseudocercospora ulei* (Guyot and Le Guen, 2018), anthracnose caused by *Colletotrichum* species (Cao et al., 2019; Liu et al., 2023), the powdery mildew caused by *Oidium heveae* (Limkaisang et al., 2005), and tapping panel dryness syndrome (TPD), the cause of which remains unknown (Ramachandran et al., 2000; Pellegrin et al., 2004).

To date, there have been four reports of natural viral infections in rubber trees, but the exact etiology of these viruses remains unconfirmed (Gama et al., 1983; Fonseca et al., 2018; Li et al., 2020; Chen et al., 2022). The first documented virus to infect rubber trees, belonging to the genus *Carlavirus* of the family *Betaflexiviridae*, was found in stunted seedlings displaying inter-veinal chlorosis, suggesting an association with leaf disease (Gama et al., 1983). Three decades later, the second putative virus named *Hevea brasiliensis* virus (HBrV) was detected in Brazil by small RNA sequencing (sRNA-seq). Phylogenetic analysis of the 1913 amino acid (aa) viral sequence assembled from HBrV showed the closest relationship to members of the genus *Maculavirus*, family *Tymoviridae*. HBrV infection was latent, as it was detected in asymptomatic plants with uniformly green leaves (Fonseca et al., 2018).

Rubber tree plantations represent a significant sector of the tropical crops industry in Hainan, China, but they suffer serious losses due to TPD. High-throughput RNA sequencing (RNA-seq) technologies have been applied to identify pathogenic microorganisms in affected plants (Wu et al., 2015). In an effort to identify potential causal agents of TPD, a comparative transcriptome analysis of TPD-affected and healthy bark samples collected from Hainan, China, led to the discovery and identification of two viruses: Rubber tree virus 1 (RTV1) and Rubber tree latent virus 1 (RTLV1), previously annotated as Cherry virus A (CVA) and Tobacco mosaic virus (TMV), respectively (Li et al., 2020; Chen et al., 2022). RTV1 is a member of the genus *Capillovirus* of family *Betaflexiviridae*, encoding two overlapping open reading frames (ORF) corresponding to a replicase-coat protein (CP) fusion polyprotein and a movement protein (MP), respectively. Complete genome of RTV1 consists of 6,811 nucleotides (nt), excluding the poly(A) tail (Li et al., 2020). Although both RTV1 and RTLV1 were identified from TPD-affected barks, infection of RTLV1 was considered to be asymptomatic, as it could be detected in 35 of 44 healthy trees. RTLV1 was suggested to be an unassigned member of the family *Virgaviridae*, as its replicase clustered with unclassified members and was not closely related to any genus of *Virgaviridae* in phylogenetic analysis. Interestingly, RTLV1 was most closely related to several arthropod-infecting viruses, with its genomic organization and length being distinct from those of plant-infecting Virgaviruses (Chen et al., 2022). RTLV1 may potentially constitute a new virus genus while its taxonomy and evolution history still remain elusive.

In this work, we identified a novel relative of RTLV1, provisionally named Rubber tree latent virus 2 (RTLV2) in co-infection with RTLV1, and confirmed their presence in commercial rubber tree plantations. The complete genome of RTLV2 was much longer than the previously reported genome of RTLV1, suggesting that the reported RTLV1 genome might be incomplete, which was re-sequenced and corrected in this work. We conducted a comparative analysis of genome organization, putative gene products, and phylogenetic relationships of the two viruses. Experimental confirmation demonstrated the mechanical transmissibility and latent infection of RTLV2.

## Materials and methods

### RNA-seq screening of viral agents in rubber tree bark samples

The RNA library construction and RNA-seq had been conducted previously (Li et al., 2020). Two bark samples used for RNA-seq analysis were collected from a TPD tree and a healthy tree, respectively, in a commercial plantation in Qionghai County, Hainan Province, China. The bark samples were stored at −80°C until used. Total RNA was isolated using TRIzol reagent (Invitrogen, China) according to the manufacturer’s instructions. The quality and purity of the extracted RNA were examined using agarose electrophoresis, a NanoPhotometer® spectrophotometer (Implen, CA, USA), and the RNA 6000 Nano Kit assay with the Agilent Bioanalyzer 2,100 system (Agilent Technologies, CA, USA). Importantly, the Ribo-Zero™ rRNA Removal Kit (Plant Leaf) was used to remove rRNA and first-strand cDNA was synthesized through reverse transcription using random hexamers so that bacterial and viral transcripts without poly(A) tails could be detected. After ligation of distinct adaptors, cDNA was purified with the Agencourt AMPure XP system (Beckman Coulter, MA, USA) to preferentially select 250 to 300 base pair (bp) fragments prior to generation of the two final libraries through PCR. Sequencing was conducted and paired-end reads were generated on the Illumina Hiseq 2,500 platform at Novogene Co. (Beijing, China). Raw reads in FASTQ format were filtered to obtain high-quality clean reads. Transcriptome assembly was accomplished using Trinity through combined short reads with a certain overlap length (*k*-mer = 25) (Grabherr et al., 2011). The assembled unigenes were clustered using default parameters (Davidson and Oshlack, 2014) and then subjected to similarity searches against the NCBI non-redundant nucleotide and protein databases (NR) and the Swissprot database for annotation and screening of viral transcripts (Altschul et al., 1997; Buchfink et al., 2015).

### Sequencing and analysis of the viral genome

To sequence the complete viral genomes of RTLV1 and RTLV2, back-to-back primer sets producing overlapping fragments which covered nearly the complete genome (Supplementary Table S1) were designed based on the assembled unigenes from RNA-seq analysis. Rapid Amplification of cDNA Ends (RACE) was carried out to confirm the 5′ and 3′ terminal sequences using the 5’/3’RACE Kit, 2nd Generation (Roche, Indianapolis, IN) according to the manufacturer’s instructions. In the initial 3’ RACE trial, total RNA was polyadenylated prior to reverse transcription using *Escherichia coli* Poly(A) Polymerase (New England Biolabs, Ipswich, MA) in case the viruses do not possess a poly(A) tail. PCR was carried out using PrimeSTAR® GXL DNA Polymerase (TaKaRa, Dalian), and the resulting products were incubated with Taq-polymerase at 72°C for 10 min then ligated into the pMD-19 T vector (TaKaRa, Dalian). At least three independent positive clones of each fragment were subjected to Sanger sequencing (Sangon Biotech, China). Overlapping sequences were analyzed and assembled to attain the consensus full-length genome with SeqMan Pro 7.1.0 (Lasergene, GATC Biotech).

ORFs and the conserved functional domains were predicted using ORF Finder^1^ and the Conserved Domain Database (CDD^2^) (Lu et al., 2020), respectively. Predicted ORFs were annotated by blast against the NCBI NR database and the Swissprot database. Potential transmembrane domains were predicted using TMHMM 2.0 (see footnote 2). Amino acid (aa) sequence identity between homologous ORFs of RTLV1 and RTLV2 as well as the remote relatives from other viruses was calculated by DNAMAN software (Lynnon BioSoft).

### Phylogenetic analysis

Representative species of all seven genera of the family *Virgaviridae* and the related taxa in orders *Martellivirales* and *Hepelivirales* were chosen to demonstrate the basic phylogenetic relationship of this virus group with alpha-like replicases. Top-scored relatives identified in blast searches, most of which are unassigned species with low homology to ratified members, were also included to identify probable virus group. Sequences were aligned using the ClustalW program implemented in the MEGA7 software with default parameters. Phylogenetic trees were then constructed in MEGA7 by using the Maximum Likelihood method based on the JTT matrix-based model with 1,000 bootstrap replications (Jones et al., 1992; Kumar et al., 2016). The calculated trees were further edited with Adobe Photoshop.^3^

### Incidence survey

Leaf samples of 44 asymptomatic rubber trees were collected from rubber tree plantations in Dingan County (Hainan Province, China) in 2020. A further collection of leaves of 30 healthy rubber trees with the same sampling standards was carried out in rubber tree plantations in Qionghai County. Total RNA was extracted with 100 mg sample using the RNAprep Pure Plant Plus Kit (TIANGEN BIOTECH, China) according to the manufacturer’s instruction. cDNA was synthesized from total RNA with random hexamer primers using the RevertAid First Strand cDNA Synthesis Kit (Thermo Fisher Scientific) following the manufacturer’s instruction. Two individual primer sets were used for detection of each virus (Supplementary Table S1). PCR conditions were 30 cycles of denaturation at 95°C for 15 s, primer annealing at 60°C for 20 s, and extension at 72°C for 2 min (2 kb product), followed by final extension at 72°C for 5 min, using the SanTaq Plus PCR Mix (Sangon Biotech, China).

### Mechanical transmission

Thirty seedlings of rubber tree and *N. benthamiana* were used for mechanical inoculation, respectively. Rubber tree seedlings (cultivar Reyan7-33-97) at the three/four true-leaf stage were purchased from the Rubber Research Institute of Chinese Academy of Tropical Agricultural Science and cultivated in greenhouse under controlled conditions of 26 ± 2°C, 16 h/8 h light/dark before used for mechanical inoculation assay. All seedlings were tested for the presence of RTLV1 and RTLV2 in groups of 10 by RT-PCR to ensure that they were uninfected prior to inoculation. *N. benthamiana* seedlings were germinated and kept in greenhouse under controlled conditions of 22 ± 2°C, 16 h/8 h light/dark. Rubber tree leaves co-infected by RTLV1 and RTLV2 were collected from Qionghai County and severed as inoculum. Fresh leaves were ground in 0.1 M phosphate buffered saline (PBS, pH 7.0) and then manually applied with carborundum powder (600 mesh) to Rubber tree or *N. benthamiana* seedlings. Mock inoculation was performed with PBS and carborundum powder. The inoculated plants were monitored for symptoms of infection and tested for presence of virus at 2 months post inoculation (mpi) by RT-PCR as described above.

## Results

### Discovery of RTLV2, a novel virga-like virus by RNA-seq

We had previously conducted a comparative transcriptome analysis of rubber tree bark samples from trees affected by TPD and healthy ones (Li et al., 2020). A total of 65,986 unigenes were *de novo* assembled with Trinity and Corset and then annotated by BLAST searches against NCBI NR and Swissprot databases. This analysis found multiple virus-like sequences, leading to the discovery and identification of RTV1 and RTLV1 (Li et al., 2020; Chen et al., 2022). Upon further examination of the assembled contigs, an interesting phenomenon were observed: aside from the RTLV1-derived unigenes that exhibited the closest similarity to the TMV replicase large subunit, several unigenes with sizes ranging from 8,211 to 8,796 nucleotides exhibited the highest similarity to the replicase large subunit of Tomato mosaic virus (ToMV). This finding strongly suggests the presence of another virga-like virus in the bark samples, as outlined in Supplementary Table S2. Reflecting the phylogenetic relationship between TMV and ToMV, the ORF1 sequences encoding the replicase from these two groups of contigs were closely related. They shared approximately 60% nt and 60% aa identity with each other. In contrast, when compared to their closest relatives in a blastx search against the GenBank NR database, they exhibited only about 35% aa identity. Given the substantial similarity in the replicase-coding sequences, we tentatively designated this newly discovered virga-like virus as RTLV2 in reference to RTLV1.

### Sequencing of the complete genomes of RTLV1 and RTLV2 by RT-PCR and RACE

Based on the alignment of the RTLV2-derived unigenes, four back-to-back primer sets for RT-PCR, nested reverse primers for 5’RACE and forward primers for 3’RACE were designed to amplify the complete genome of RTLV2 (Supplementary Table S1A). Expected bands of three primer sets with sizes of approximately 2 kilobases (kb) were obtained, which covered the 5′ majority of the unigenes with overlaps between adjacent fragments. 5’RACE with different combinations of nested primers produced unique clear bands corresponding to the expected sizes, and an identical 5′ end was obtained from the 20 clones sequenced. However, the fourth primer set covering the 3′ 2 kb region of the unigenes generated a large band of nearly 6 kb, which was approximately 3.5 kb longer than expected. Moreover, 3’ RACE produced multiple bands forming gradient ladders in the serial nested amplifications, suggesting the random breakage near the 3′ end of viral RNA. We therefore speculated that the assemble unigenes contained a 3.5 kb missing gap near the 3′ end. Based on the sequence of the 6 kb band, additional primers inside and outside of the presumed gap were designed. RT-PCR with various combinations of primers confirmed the fidelity and continuity of the 6 kb amplicon as authentic viral sequence. A second trial of 3’ RACE with nested forward primers (Supplementary Table S1A) located at the 3′ proximal of the 6 kb sequence produced unique clear bands corresponding to the nested primers, and an identical 3′ end was obtained from the 10 clones sequenced. 3’RACE using total RNA or 3′-polyadenylated RNA gave the same results, confirming the existence of poly(A) tail at the 3′ end. Sequences of all the amplicons were assembled to generate the complete genome sequence of RTLV2 (GenBank accession OR500511), which turned out to be 11,866 nt in length, encoding seven ORFs.

The RTLV2 genome is much longer than the previously determined the genome sequence of RTLV1, which consists of 9,422 nt (GenBank accession MW774645) (Chen et al., 2022). Due to the close genetic correlation between RTLV1 and RTLV2, namely approximately 60% nt and 60% aa identities, it is reasonable to speculate that the previously determined RTLV1 genome is incomplete. Given that the assembled RTLV2-derived unigenes contained a 3.5 kb missing gap, it is possible that similar error occurred in the transcriptome analysis pipeline which generates 3′-truncated RTLV1 contigs. To verify the 3′ end of RTLV1, 3’RACE was conducted with the 72°C extension process extended to 3 min per amplification cycle. In accordance with this assumption, serial bands of about 3 kb were obtained in serial amplifications with various combinations of nested primers which located within 1 kb upstream of the reported RTLV1 3′ end. Based on the consensus sequence of these 3 kb products, five forward nested primers near the 3′ terminal were designed to conduct a second trial of 3’RACE (Supplementary Table S1B), still with the 72°C extension process extended to 3 min per amplification cycle for fully extension. Unique clear bands of about 0.5 kb corresponding to the nested primers were obtained, and a uniform 3′ end was identified from the 10 clones sequenced. 3’RACE with total RNA excluding the polyadenylation procedure gave the same results, confirming the existence of poly(A) tail at the 3′ end. The rest of RTLV1 genome was fully re-sequenced by RT-PCR of overlapping fragments and 5’ RACE, and sequences of all the amplicons were assembled to generate the complete genome sequence of RTLV1 (GenBank accession OR500510), which turned out to be 11,923 nt in length, excluding the poly(A) tail.

### Genome annotation of RTLV1 and RTLV2

**Figure 1:**
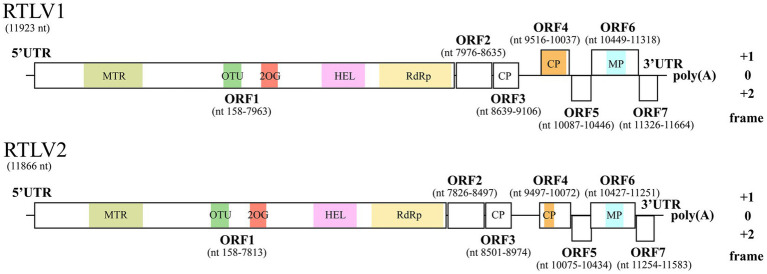
Genome organization of RTLV1 and RTLV2. RTLV1 and RTLV2 are closely related and have similar genomic architecture encoding seven pairs of ORFs. Conserved domains predicted by CDD were colored and labeled as: MTR, methyltransferase (pfam01660); OTU, ovarian tumor superfamily (cl45892); 2OG, 2OG-Fe(II) oxygenase superfamily (cl21496); HEL, RNA helicase (pfam01443); RdRp, RNA-dependent RNA polymerase (pfam00978); CP, TMV-like coat protein (pfam00721); MP, viral movement protein (pfam01107). Two uncolored putative CPs coded by ORF3 were not recognized by CDD, but shared conserved aa residues with the CDD-predicted CP coded by ORF4 and CPs of Virgaviruses (Figure 2). Genomic position of each putative ORFs were drawn to scale, and the reading frame of ORFs were determined relative to ORF1 (frame 0). UTR, untranslated region.

RTLV1 and RTLV2 shared 58.31% identity of the genome nt sequences. Both genomes had a G/C content of 39% and were predicted to encode seven ORFs which displayed similar layout of genome organization (Figure 1). All ORFs shared marginal (30.36% for ORF7) to considerable (64.17% for ORF5) aa identity with their homologue encoded by the other virus, demonstrating the close genetic relationship between RTLV1 and RTLV2 (Table 1).

**Table 1:** ORFs and their putative gene products encoded by RTLV1 and RTLV2.

ORF	Amino acids (aa)	MW (kDa)	Shared nt identity (%)	Shared aa identity (%)	Protein annotation	Similarity search against the NCBI NR database						
						Best matched plant virus	Classification	Host/origin	Protein accession	Query cover (%)	aa identity (%)	*E*-value
RTLV1 ORF1	2,601	298.2	60.75	58.87	Replicase polyprotein	Oxera neriifolia associated virus	Unclassified	*Oxera neriifolia*	CAI5383846	71	37.89	0.0
RTLV2 ORF1	2,551	291.3				Oxera neriifolia associated virus	Unclassified	*Oxera neriifolia*	CAI5383846	73	38.66	0.0
RTLV1 ORF2	219	24.5	52.83	42.60	Unknown	Oxera neriifolia associated virus*	Unclassified	*Oxera neriifolia*	CAI5383847	NA	NA	NA
RTLV2 ORF2	223	25.0				Oxera neriifolia associated virus	Unclassified	*Oxera neriifolia*	CAI5383847	61	29.71	2E-03
RTLV1 ORF3	155	17.8	59.49	56.69	Coat protein	Oxera neriifolia associated virus*	Unclassified	*Oxera neriifolia*	CAI5383848	NA	NA	NA
RTLV2 ORF3	157	18.1				Oxera neriifolia associated virus*	Unclassified	*Oxera neriifolia*	CAI5383848	NA	NA	NA
RTLV1 ORF4	173	19.6	50.91	52.00	Coat protein	Plant associated tobamo-like virus 1	Unclassified (*Virgaviridae*)	*Solanum lycopersicum*	UTQ50505	85	28.86	6E-04
RTLV2 ORF4	191	22.0				Goji berry chlorosis virus	Unclassified	*Lycium chinense*	AYO99570	78	28.76	1E-04
RTLV1 ORF5	119	13.0	65.29	64.17	Unknown	Oxera neriifolia associated virus*	Unclassified	*Oxera neriifolia*	CAI5383850	NA	NA	NA
RTLV2 ORF5	119	12.8				Oxera neriifolia associated virus*	Unclassified	*Oxera neriifolia*	CAI5383850	NA	NA	NA
RTLV1 ORF6	289	32.5	60.57	60.00	Movement protein	*Pinus nigra* virus 1	Unclassified (*Caulimoviridae*)	*Pinus nigra*	AXB54820	54	34.36	3E-10
RTLV2 ORF6	274	30.4				*Pinus nigra* virus 1	Unclassified (*Caulimoviridae*)	*Pinus nigra*	AXB54820	40	33.04	2E-06
RTLV1 ORF7	112	12.4	47.49	30.36	Unknown	No matches	NA	NA	NA	NA	NA	NA
RTLV2 ORF7	109	12.2				No matches	NA	NA	NA	NA	NA	NA

**Figure 2:**
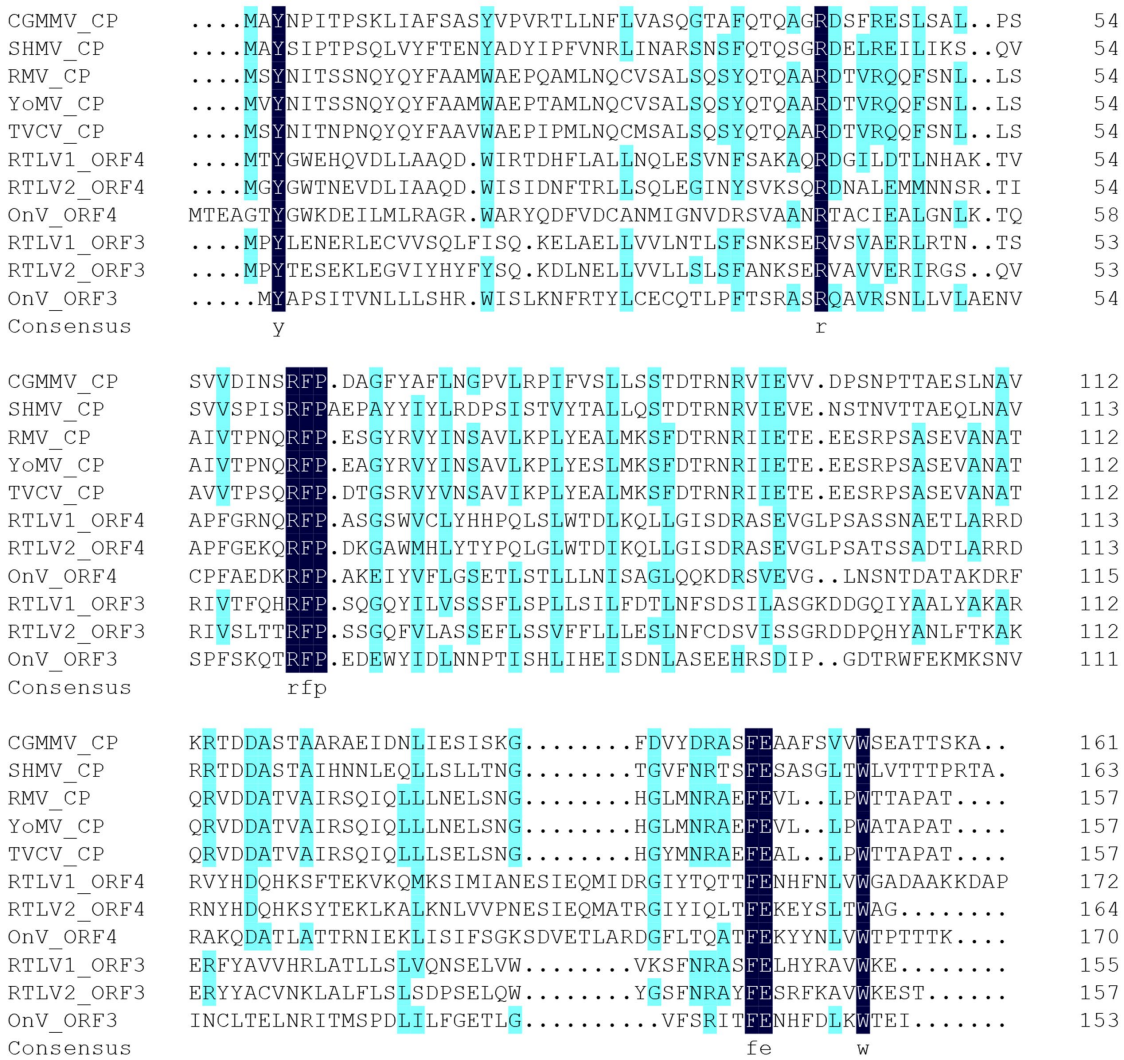
Amino acid sequence alignment of the putative CPs of RTLV1, RTLV2, OnV and reviewed CPs of Tobamoviruses. Identical residues were highlighted by dark blue, including two motifs RFP and FE which extensively exist in the TMV-like CP domain family (pfam00721). aa residues with homology level ≥ 50% were highlighted by light blue (calculated by DNAMAN). Virus species and the Swissprot accession of reviewed CPs of assigned members of genus *Tobamovirus*, family *Virgaviridae* are as follows: CGMMV, Cucumber green mottle mosaic virus, P69475; SHMV, Sunn-hemp mosaic virus, P03581; RMV, Ribgrass mosaic virus, Q9WDG7; YoMV, Youcai mosaic virus, Q66222; TVCV, Turnip vein-clearing virus, Q88922. OnV, Oxera neriifolia associated virus. aa sequences of OnV ORF3 and ORF4 were translated from nt 8,695–9,156 and nt 9,222–9,734 of the full-length genome (GenBank accession OX380366), respectively.

The best matched plant virus for each ORF was determined by blastx max scores in search against the NCBI NR database, except for items marked with an asterisk (*), that were inferred by sequence alignment of aa sequences as no significant results returned. (Refer to Supplementary Figures S1, S2 and Figure 2 for ORF2, ORF3, and ORF5, respectively.) CDS, coding sequence; MW, molecular weight; NA, not available (no returned significant hits).

ORF1 in RTLV1 and RTLV2 shares a nucleotide identity of 60.75% and an amino acid identity of 58.87%. This ORF encodes a substantial replicase polyprotein, weighing 298.2 kDa for RTLV1 and 291.3 kDa for RTLV2. It encompasses three conserved domains predicted by the Conserved Domain Database (CDD) (see Figure 1 and Table 1): a viral methyltransferase domain (MTR, pfam01660), a viral RNA helicase domain (HEL, pfam01443) and an RNA-dependent RNA polymerase domain (RdRp, pfam00978). The predicted MTR domain is the hallmark of the ‘Sindbis-like’ supergroup of positive-strand RNA viruses, such as Hordei-, Tobra-, Tobamo-, Bromo- and Closteroviruses (Rozanov et al., 1992). The 800 nt helicase domain contained the conserved NTP-binding motifs GKT and DE (Koonin and Dolja, 1993), which were identified from aa 1789–1791 and 1873–1874 of RTLV1 ORF1, and aa 1738–1740 and 1821–1822 of RTLV2 ORF1, respectively. The well-characterized GDD motif in the RdRp domain (Koonin, 1991) was found from aa 2,452–2,454 of RTLV1 ORF1 and aa 2,402–2,404 of RTLV2 ORF1. In addition to the domains conservatively associated with viral replication, a 2OG-Fe(II) oxygenase superfamily (CDD accession cl21496) (Aravind and Koonin, 2001) and an ovarian tumor (OTU) superfamily (CDD accession cl45892) (Messick et al., 2008) were found in the intergenic region between MTR and HEL (Figure 1), with lower *E*-value of about 1e-10 to 1e-12. Blastx against NCBI NR database showed that ORF1 best matched the replicase polyprotein of Oxera neriifolia associated virus (OnV), an unclassified plant virus found in large-scale transcriptome data mining (Mifsud et al., 2022; Table 1). However, the rest of the top-scored hits were basically replicase proteins encoded by unclassified invertebrate viruses followed by replicases of Tobamoviruses (Supplementary Table S3), indicating that RTLV1 and RTLV2 were virga-like viruses related to the family *Virgaviridae*.

RTLV1 ORF2, with a molecular weight of 24.5 kDa, and RTLV2 ORF2, weighing 25.0 kDa, exhibited a shared nucleotide identity of 52.83% and an amino acid identity of 42.60%. Despite our analyses with the Conserved Domain Database (CDD), we were unable to identify any conserved domains within ORF2. Blastx against NR revealed marginal homology between RTLV2 ORF2 and the putative ORF2 of OnV, which encodes a replicase polyprotein closely related to RTLVs ORF1 (Table 1). Additionally, Blastp analysis of RTLV2 ORF2 against the Swissprot database revealed only one distant viral homologue (with an amino acid identity of 25.4%), which was the putative ORF2 of Hubei virga-like virus 11 (HVLV11), an unclassified invertebrate virus identified through high-throughput transcriptome data mining (Shi et al., 2016). Although we pursued similar investigations for RTLV1 ORF2, we could not identify any significant hits. However, upon conducting multiple alignments (please see Supplementary Figure S1), we did identify several conserved amino acid residues within the C-terminal region of ORF2 in RTLV1, RTLV2, OnV, and HVLV11. This finding suggests a potential common origin for these proteins, which currently have unknown functions, and implies a distant phylogenetic relationship among these unclassified viruses.

RTLV1 ORF3, with a molecular weight of 17.8 kDa, and RTLV2 ORF3, weighing 18.1 kDa, shared a nucleotide identity of 59.49% and an amino acid identity of 56.69%. Standard Blastx analysis against the NCBI NR database and searches in the CDD did not yield significant hits for ORF3. However, Blastp analysis against the Swissprot database unveiled Tobamovirus coat protein (CP)-like motifs in both RTLV1 and RTLV2 ORF3, suggesting that ORF3 likely encodes a viral CP. Despite the ambiguous annotation of ORF3 as a CP, RTLV1 ORF4, with a molecular weight of 19.6 kDa, and RTLV2 ORF4, weighing 22.0 kDa, both contained a CP domain resembling that of Tobacco mosaic virus (TMV), as predicted by CDD (pfam00721). Blastx analysis against the NCBI NR database returned multiple hits of putative CP proteins encoded by various invertebrate virga-like viruses, as well as two unclassified plant viruses. Notably, RTLV1 ORF4 and RTLV2 ORF4 both exhibited the highest similarity to the putative CP of *Culex pipiens*-associated Tunisia virus (CpATV) (Bigot et al., 2018). Among the homologous proteins encoded by plant viruses, RTLV1 ORF4 matched the putative CP of Plant-associated tobamo-like virus 1 (PaToLV1) (Rivarez et al., 2023), while RTLV2 ORF4 matched the putative CP of Goji berry chlorosis virus (GBCV) (Table 1; Supplementary Table S4).

Given that both ORF3 and ORF4 contain motifs or domains resembling the TMV CP, we conducted a multiple sequence alignment involving ORF3 and ORF4 encoded by RTLV1, RTLV2, OnV, and several Tobamovirus-encoded CPs. This analysis identified two common motifs, RFP and FE, which are abundant in the TMV-like CP domain family pfam00721 (refer to Figure 2). However, despite the presence of these common CP-like motifs and some conserved amino acid residues in the C-terminal region, ORF3 and ORF4 exhibited extremely low amino acid identities of 18.50% for RTLV1 and 17.47% for RTLV2. Between the putative CPs encoded by ORF3 and ORF4, we identified a large intergenic region spanning 409 nt for RTLV1 and 522 nt for RTLV2 (refer to Figure 1). Within this region, ORF Finder recognized two small ORFs of 46 amino acids (nt 9,103–9,243) and 58 amino acids (nt 9,230–9,406) in RTLV1, and three small ORFs of 41 amino acids (nt 8,998–9,123), 63 amino acids (nt 9,092–9,283), and 50 amino acids (nt 9,287–9,439) in RTLV2. However, these ORFs appear to be hypothetical, as no significant homology resembling the pairs of ORF1-7 was detected.

Among the seven pairs of ORFs, ORF5 encoded the most conserved protein between RTLV1 and RTLV2, sharing 65.29% nt and 64.17% aa identities (Table 1). When Blastp analysis was conducted against the Swissprot database, limited similarity with a few proteins containing kinase domains from plants and transmembrane proteins from bacteria was observed. However, this similarity only covered about 15 to 30% of the sequence. In contrast, Blastx analysis against the NCBI NR database failed to yield any significant hits. It’s noteworthy that ORF5 was identified as the sole probable transmembrane protein according to TMHMM predictions, with relatively low probabilities (approximately 0.6, 0.7, and 0.2) assigned to three potential transmembrane regions. Similar to ORF2, which exhibited no apparent homology with known proteins and may be specific to viruses within their clade, our alignment of the amino acid sequences of ORF5 from RTLV1, RTLV2, and OnV revealed several conserved residues, indicating their phylogenetic relationship (please refer to Supplementary Figure S2).

Moving on, RTLV1 ORF6 (32.5 kDa) and RTLV2 ORF6 (30.4 kDa) encoded the largest protein excluding the replicase polyprotein. CDD analyses identified a viral movement protein domain (pfam01107) in the center region of ORF6 (Figure 1). Blastx against NR revealed the most related viral homologue of ORF6 was the putative MP of *Pinus nigra* virus 1 (Rastrojo et al., 2018) (*Caulimoviridae*, Table 1). Blastp against Swissprot returned hits of viral MPs from families *Caulimoviridae*, *Betaflexiviridae,* and *Rhabdoviridae*. pfam01107 also includes MPs from the family *Virgaviridae*, e.g., the “30 K” MP of TMV and Tobacco rattle virus (TRV) (Melcher, 2000), which have similar molecular weight as ORF6. However, significant hits of members from *Virgaviridae* were not found in blast searches, suggesting that their homology with ORF6 was limited.

Finally, RTLV1 ORF7 (12.4 kDa) and RTLV2 ORF7 (12.2 kDa) encoded the smallest protein in the viral genome. Blast searches found no apparent homology with known proteins and no conserved domains were identified. Interestingly, ORF7 was the most divergent in the seven pairs of ORF, and the 30.36% aa identity was much lower than the 47.49% nt identity shared between ORF7 of RTLV1 and RTLV2, suggesting a species-specific differentiation.

### Phylogenetic analysis

**Figure 3:**
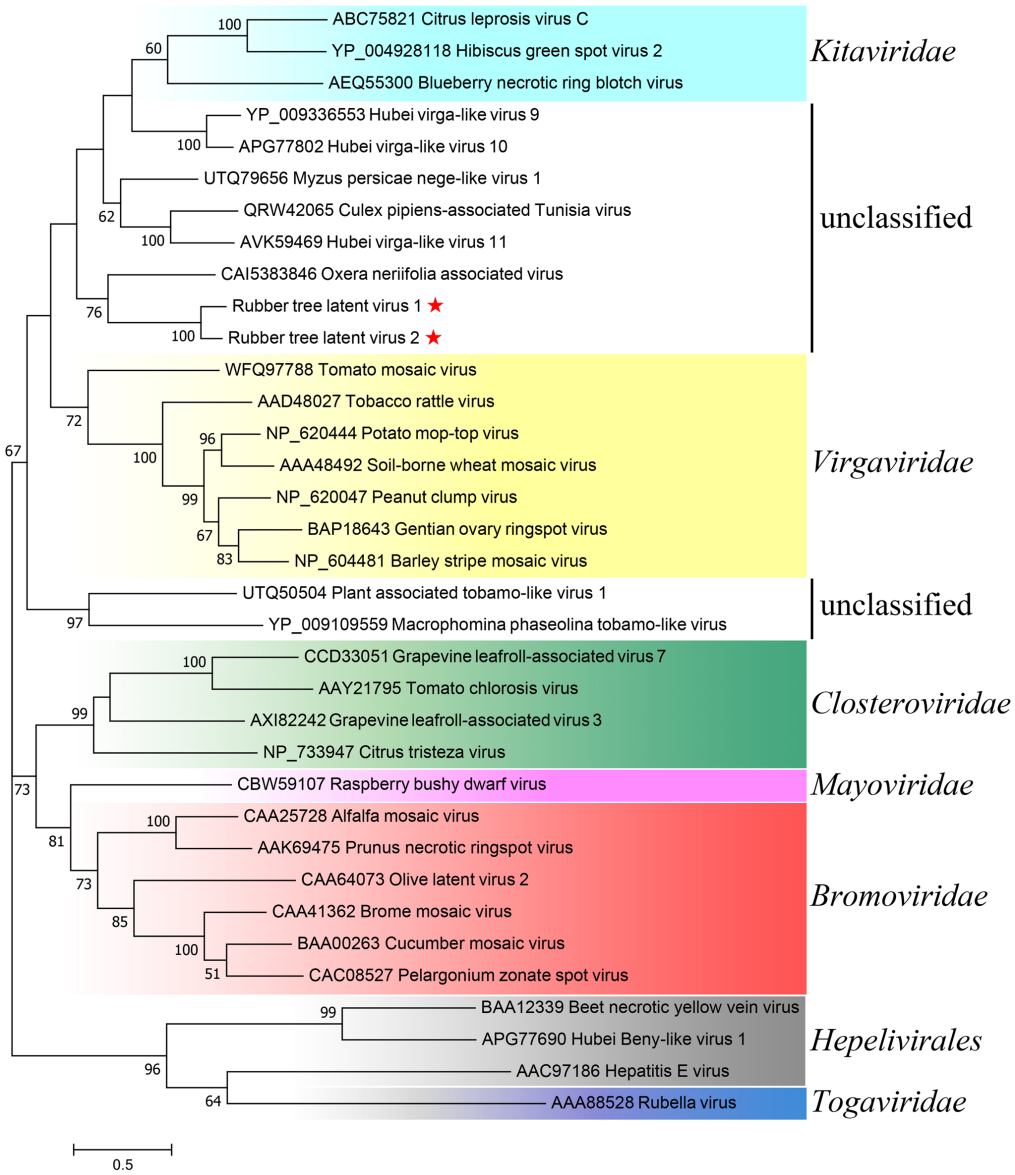
Phylogenetic analysis of RTLV1 and RTLV2 with representative members of *Virgaviridae*, *Virgaviridae*-related taxa, and unclassified virga-like viruses based on the aa sequences of the RdRp domain. Evolutionary analyses were conducted in MEGA7 by using the Maximum Likelihood method based on the JTT matrix-based model (Jones et al., 1992; Kumar et al., 2016). The tree with the highest log likelihood (−27619.36) was shown. The percentage of trees in which the associated taxa clustered together is shown next to the branches (where ≥50%). Branch lengths were measured in the number of substitutions per site. Representative species of the major genera of families *Virgaviridae*, *Bromoviridae*, *Closteroviridae*, *Kitaviridae*, and *Mayoviridae* (order *Martellivirales*) as well as the order *Hepelivirales* were used to present the current classification schemes of viruses with alpha-like replicases (Adams et al., 2017; Mifsud et al., 2022). GenBank accessions were noted before the virus name.

Preliminary Blastx search of ORF1 (the replicase polyprotein) against NR found homologues encoded by unclassified invertebrate- or plant-infecting virga-like viruses and assigned members from genus *Tobamovirus* of family *Virgaviridae* (Supplementary Table S3). However, the majority of homologues found in Blastx of ORF4 containing the TMV CP-like domain were putative CPs of unclassified invertebrate-infecting viruses, while the CPs of ratified members of *Virgaviridae* were absent among the returned hits (Supplementary Table S4). To investigate the taxonomic position of RTLV1 and RTLV2, aa sequences of the RdRp domain of representative species from *Virgaviridae*, *Virgaviridae*-related taxa (Adams et al., 2017) and unclassified viruses with homologous replicase found in Blastx were used in phylogenetic analysis (Figure 3). The generated tree conformed to the general phylogenetic topology of *Virgaviridae* with its related taxa in orders *Martellivirales* and *Hepelivirale* (Adams et al., 2017), while the RdRp of unclassified viruses formed two clusters in the *Kitaviridae*-*Virgaviridae* branch. Consistent with the Blastx results (Supplementary Table S3), RTLV1 and RTLV2 clustered with OnV and formed a monophyletic clade separated from the invertebrate viruses. This clade could form a paraphyletic group together with the *Virgaviridae* branch, however the node support was only moderate (42%) for this subjective partition.

**Figure 4:**
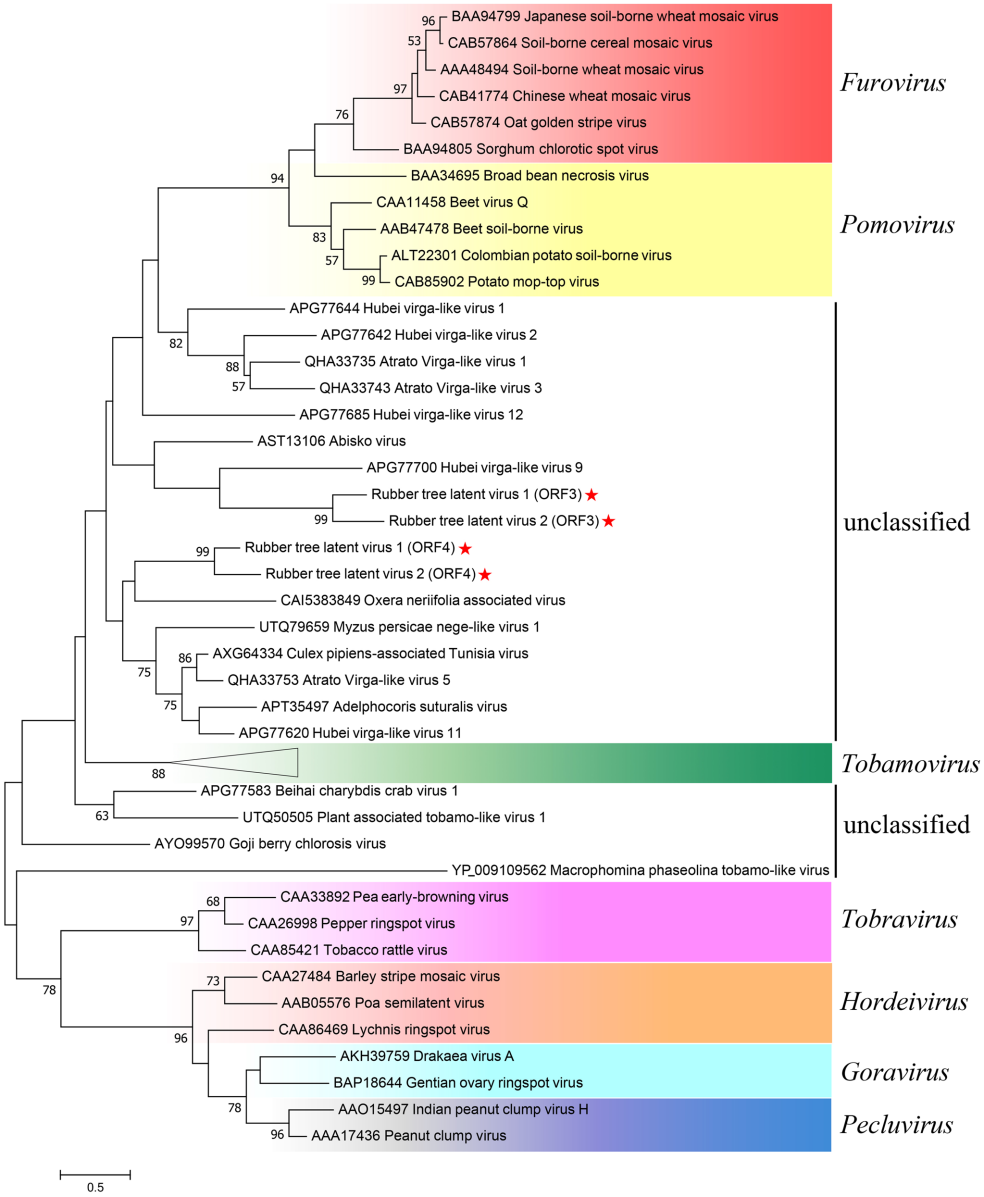
Phylogenetic analysis of RTLV1 and RTLV2 with representative members of *Virgaviridae* and unclassified virga-like viruses based on the aa sequences of the coat protein. Evolutionary analyses were conducted in MEGA7 by using the Maximum Likelihood method based on the JTT matrix-based model (Jones et al., 1992; Kumar et al., 2016). The tree with the highest log likelihood (−12162.23) was shown. The percentage of trees in which the associated taxa clustered together is shown next to the branches (where ≥50%). Branch lengths were measured in the number of substitutions per site. Representative species of the seven genera of *Virgaviridae* were used to present the current classification schemes within the family (Adams et al., 2017; Bigot et al., 2018). GenBank accessions were noted before the virus name. The subtree of Tobamoviruses contained 13 representative members including TMV, ToMV, etc. (GenBank accessions: AEV53623, AEW50167, AAN51974, AEF01561, BAA02071, BAF37647, ADN93256, AIA57393, AAC02785, BAC07269, AAB02337, NP_597750, AAK17991) and was compressed to present the whole figure on one page.

As ORF3 and ORF4 both contained TMV CP-like motifs (Figure 2), aa sequences of CPs of representative species from *Virgaviridae* and CP-like homologues found in Blastx were used to investigate the phylogenetic relationship of RTLV1 and RTLV2 with *Virgaviridae* (Figure 4). CPs from the seven genera of *Virgaviridae* clustered in a genus-specific way, forming distinct monophyletic or paraphyletic groups reconstructing the CP phylogenetic topology within *Virgaviridae* (Adams et al., 2017). Most CPs of unclassified viruses were placed between the *Tobamovirus* and *Furovirus*-*Pomovirus* clades and could be divided into two groups which contained ORF3 and ORF4, respectively. Nevertheless, bootstrap support for most nodes in this heterogeneous group was low, probably due to the lack of evolutionary links between them. Phylogenetic relationship based on the RdRp domain suggested a common evolutionary origin with families of the order *Martellivirales*, within which RTLV1 and RTLV2 may potentially constitute a new virus family.

### Virus prevalence in rubber tree plantations

A limited survey of prevalence of RTLV1 and RTLV2 was conducted in rubber tree plantations in Qionghai County and Dingan County of Hainan, China. We have previously detected RTLV1 in 35 out of 44 asymptomatic trees collected from Dingan County (Chen et al., 2022). Using the same sample pool, RTLV2 was detected in 32 trees by RT-PCR with two primer sets (Supplementary Table S1A). A second batch of 30 trees showing no viral symptom were collected from Qionghai County and analyzed following the same protocol. One tree and four trees tested positive for RTLV1 and RTLV2, respectively. Most remarkably, a high rate of co-infection of the two viruses was observed (Table 2). In the Dingan samples pool, 32 trees (91.4%) out of 35 RTLV1-infected trees were co-infected by RTLV2, and all 32 RTLV2-infected trees were co-infected by RTLV1.

**Table 2:** Limited incidence survey of RTLV1 and RTLV2 in commercial plantations in Hainan, China.

Sampling location	Sample size	Uninfected	Infected by RTLV1	Infected by RTLV2	Infected by RTLV1 only	Infected by RTLV2 only	Co-infected by RTLV1 and RTLV2
Dingan	44	9	35	32	3	0	32
		(20.5%)	(79.5%)	(72.7%)	(6.8%)	(0.0%)	(72.7%)
Qionghai	30	26	1	4	0	3	1
		(86.7%)	(3.3%)	(13.3%)	(0.0%)	(10.0%)	(3.3%)

Two batches of 44 and 30 healthy trees showing no viral symptom and continually producing latex were collected from Dingan and Qionghai County of Hainan, respectively, for RT-PCR detection of the presence of RTLV1 and RTLV2. Frequent co-infection of the two viruses was observed in the Dingan sample pool. The percentage within brackets was calculated relative to the sample size of each location.

### Mechanical transmissibility assay

**Figure 5:**
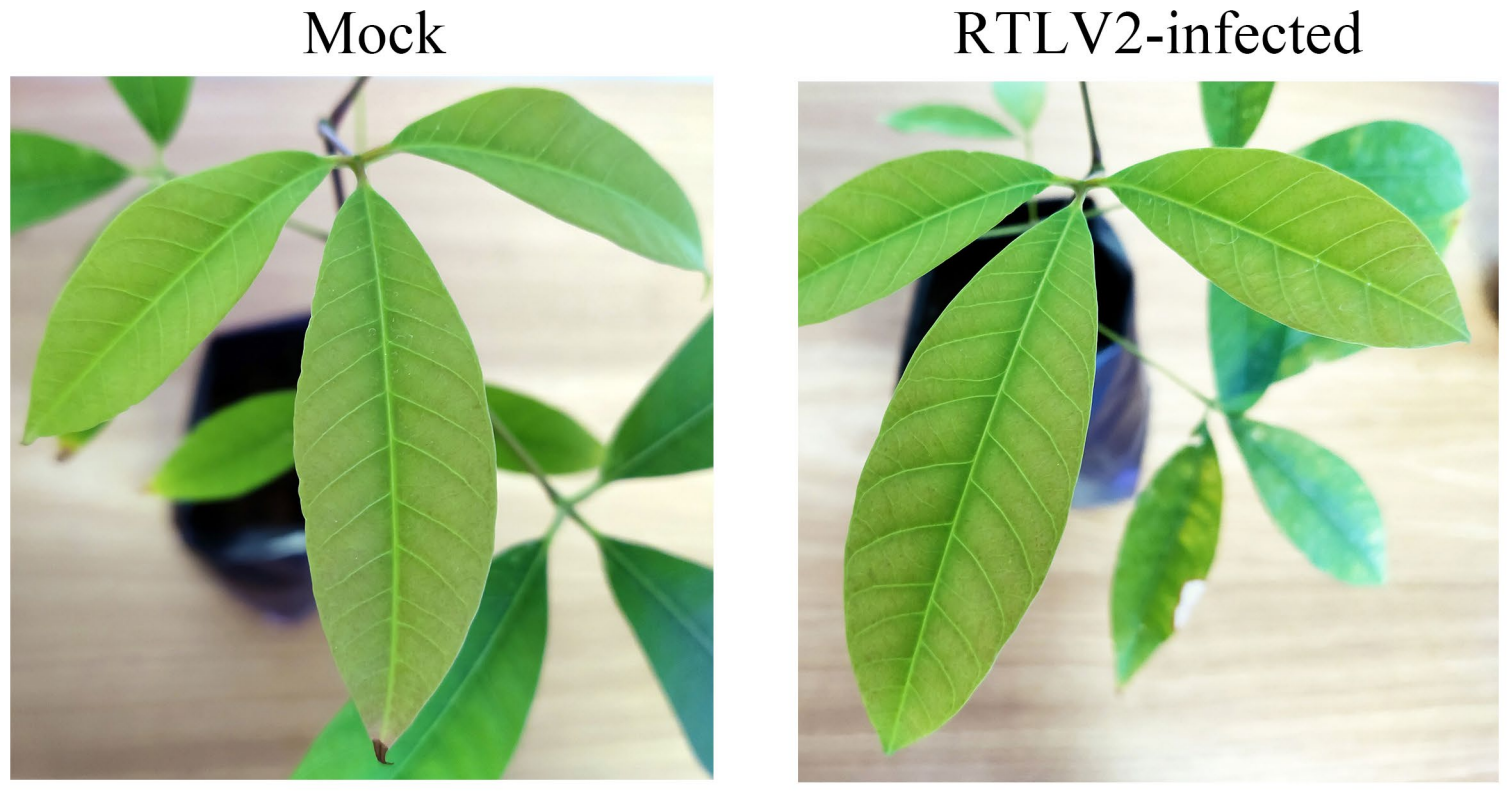
Latent infection of RTLV2 in rubber tree seedlings at 2 mpi. Homogenates of fresh leaves co-infected by RTLV1 and RTLV2 was mechanically inoculated onto rubber tree seedlings while mock-inoculation of PBS severed as control (Mock). RTLV2 was detected by RT-PCR in the upper systemic leaves which showed no visible symptoms. RTLV1 failed to transmit under the same condition.

Mechanical inoculation of RTLV1 and RTLV2 was carried out using homogenates of fresh leaves co-infected by the two viruses. Thirty seedlings of rubber trees tested virus-free and *N. benthamiana* were manually inoculated and continuously observed until detection at 2 mpi, respectively. No visible difference or viral symptom was observed amongst plants inoculated with viruliferous inoculum and mock-inoculated control plants. Top systemic leaves were sampled for RT-PCR detection and all 30 *N. benthamiana* plants remained uninfected at 2 mpi. Eighteen out of 30 (60%) rubber tree seedlings tested positive of RTLV2, while no plants tested positive of RTLV1. The presence of RTLV2 was further confirmed by Sanger sequencing of the RT-PCR amplicons, which only had a few single nucleotide polymorphisms (SNP) compared with the consensus genome. Close-up observation of newly emerged leaves of RTLV2-infected plants and mock-inoculated plants confirmed the latent infection of RTLV2 in rubber tree seedling (Figure 5).

## Discussion

The TPD syndrome of rubber trees has been reported for over a century (Chandler, 1922). Numerous studies have proposed TPD as a physiological syndrome (Gebelin et al., 2013; Zhang et al., 2017). However, the search for the causal agent of TPD has persisted. While the identification of TPD-related pathogens has not yielded conclusive results, investigations have unveiled several novel life forms within the rubber tree, including viroids and previously unidentified viruses (Ramachandran et al., 2000; Pellegrin et al., 2004; Li et al., 2020; Chen et al., 2022). Among these, RTLV1 and RTLV2 are two closely related viruses that have been rarely reported in plants and potentially belong to a novel viral family.

Our current understanding of virus biodiversity and classification is limited and fragmented because virology studies have primarily concentrated on disease-causing agents (Shi et al., 2016). Extensive transcriptome mining across multiple phyla has uncovered hundreds of novel viruses that infect animals and plants, some of which exhibit unique features, potentially leading to the establishment of new virus families (Shi et al., 2016; Mifsud et al., 2022). The genomes of RTLV1 (11,923 nt) and RTLV2 (11,866 nt) exhibit a similar organization, distinct from the known plant virus families. ORF1 encompasses a viral MTR, HEL, and RdRp domain, conservatively arranged in *Virgaviridae* and related viruses with alpha-like replicases (Koonin and Dolja, 1993). The presence of a 2OG-Fe(II) oxygenase domain and an OTU-like domain between MTR and HEL (Figure 1) unmistakably places RTLV1 and RTLV2 in the Hepe-Virga virus group (Shi et al., 2016; Kondo et al., 2019). However, ORF3 and ORF4 were predicted to encode dual TMV-like CPs, contrary to the sole CP in ratified members of *Virgaviridae* (Figures 2, 4). Phylogenetic analysis showed the distinct origins for ORF3 and ORF4 that might be derived from independent acquisitions. The divergence event of RTLV1 and RTLV2 might occur after acquisition of the second CP. Parallel acquisition of multiple copies of structural proteins by the Hepe-Virga viruses is not uncommon, some of which contain triple CPs (Shi et al., 2016). The CP duplex excludes RTLV1 and RTLV2 from assignment to current genera of *Virgaviridae*. Furthermore, members of *Virgaviridae* have no poly(A) tails and usually possess a tRNA-like at the 3′-terminus, while RTLV1 and RTLV2 have a poly(A) tail.

RTLV1 and RTLV2 diverged from current plant virus families and were clustered together with OnV in phylogenetic analyses based on RdRp amino acid sequences (Figure 3). OnV was proposed to constitute a new virus family as its RdRp formed a distinct and well-supported outgroup to related plant virus families (Mifsud et al., 2022). The OnV genome is quite similar to those of RTLV1 and RTLV2 in terms of structure and phylogeny of coding proteins as all of the five ORFs encoded by OnV were homologous to corresponding ORFs of RTLV1 and RTLV2. The assembled genome of OnV has not been verified by RACE. Nevertheless, OnV is the closest relative of RTLV1 and RTLV2 among currently recorded viruses, and together they may represent the existence of a novel, uncharacterized virus group.

High-throughput sequencing pipelines typically produce fragmented viral sequences, sometimes even recombined, partially due to low transcript abundance and intrapopulation viral diversity (Studholme, 2012; Rose et al., 2016). CpATV and HVLV11, two unclassified virga-like viruses, clustered in the same clade with RTLV1 and RTLV2, were initially reported in a highly truncated form. These reports were later revised with substantial genome extensions of approximately 4 kb (Shi et al., 2016; Debat, 2017; Bigot et al., 2018; Debat and Ribeiro, 2018). In this study, the Trinity assembly of clean reads generated multiple fragments for RTLV1, RTLV2, and RTV1 (Supplementary Table S2). These fragments served as templates for designing primers used in the amplification of complete genomes. However, the assembled sequences of RTLV1 and RTLV2 contained a missing gap of 3 kb near the 3′ end. This gap led to the previously reported truncated RTLV1 genome (Chen et al., 2022). In the initial RACE experiments aimed at verifying the 3′ end of RTLV1, the 72°C extension was set at 1 min per cycle because the primers were predicted to be within 1 kb upstream of the genomic end. However, this insufficient extension generated amplicons derived from broken ends rather than the intact genomic end. The incomplete genome of RTLV1 was corrected in the present work as the missing gap in assembled sequences was found, and RACE experiments were adjusted accordingly. It is worth noting that similar truncated ends were found in the initially reported genomes of CpATV and HVLV11, which are highly AT-rich regions (Debat and Ribeiro, 2018). In the case of RTLV1 and RTLV2, the upstream of ORF4 and the 3’ UTR were AT-rich regions that were mistakenly joined in some of the assembled sequences, resulting in a large missing gap of 3 kb containing ORF4 to ORF7.

Apart from OnV, the rest of the close relatives of RTLV1 and RTLV2 revealed by phylogenetic analyses were primarily unclassified invertebrate viruses (Figures 3, 4). To exclude potential contamination, a transmissibility assay was conducted, in which RTLV2 was successfully inoculated into rubber tree seedlings (Figure 5), indicating that RTLV2 is indeed an infectious plant virus. A limited field survey of 44 and 30 trees collected from two counties confirmed the natural occurrence of RTLV1 and RTLV2, with a high rate of co-infection of the two viruses observed. This co-infection could be a sign of synergistic virus-virus interactions. However, RTLV1 failed to transmit to rubber tree seedlings using an inoculum of homogenates of fresh leaves co-infected by the two viruses, possibly due to an incompatible inoculation buffer or native features of the viruses. Mixed infections of plant viruses are common in nature and lead to various intrahost virus-virus interactions. Mixtures of synergistic and antagonistic interactions, often resulting in unpredictable biological and epidemiological consequences, are likely to occur in plants (Zhang et al., 2001; Syller, 2012). The virus-virus interaction or association between RTLV1 and RTLV2 requires further detailed studies.

At present, breeding approaches of rubber trees for latex yield improvement is restrained by the lack of efficient gene transforming methods for rubber trees (Priyadarshan, 2017). Viruses have been developed as vector tools for virus-induced gene silencing (VIGS) or gain-of-functions for many economic crops (Pasin et al., 2019; Wang et al., 2020). Tobacco rattle virus (TRV)-derived vector was reported to cause VIGS in rubber tree seedlings (Li et al., 2021), but the work has not been repeated. Application of viral vectors in perennial woody crops is still facing great challenge due to the common instability of foreign inserts in long-term trials (Dawson and Folimonova, 2013; Ding et al., 2018). RTLV1 and RTLV2 have the potential to be developed as viral vector tools and possess advantages over cross-host viral vectors as they are environmentally native and have no pathogenic effect on rubber trees.

In summary, we have characterized two novel virga-like viruses with distinct genome architectures incompatible with hallmarks of current virus families, and confirmed their natural occurrence in cultivated rubber trees. Our findings contribute to the knowledge of viral diversity in rubber trees and lay the groundwork for developing viral vector tools for rubber tree breeding and functional genomics.

## Data availability statement

RNA seq raw data are available through the central BioProject database at NCBI under project accession PRJNA1040157 and BioSamples accessions SAMN38246073, SAMN38246086, SAMN38246087, and SAMN38246088. The complete genome sequences of RTLV1 and RTLV2 are available at GenBank under accessions OR500510 and OR500511.

## Author contributions

RZ: Data curation, Formal analysis, Investigation, Writing – original draft, Writing – review & editing. XS: Data curation, Formal analysis, Investigation, Writing – review & editing. FY: Investigation, Writing – review & editing. ZL: Supervision, Writing – review & editing. XH: Funding acquisition, Project administration, Resources, Supervision, Writing – review & editing.

## Footnotes

^1^https://www.ncbi.nlm.nih.gov/orffinder/

^2^https://www.ncbi.nlm.nih.gov/Structure/cdd/wrpsb.cgi

^3^https://www.adobe.com
