# Supplementary material for: Identification and characterization of two closely related virga-like viruses latently infecting rubber trees (Hevea brasiliensis)
Source: Front Microbiol. 2023 Dec 14;14:1286369. doi: 10.3389/fmicb.2023.1286369 (PMC10752949; doi:10.3389/fmicb.2023.1286369)
Supplement: Supplementary file 1 [file Table_1.docx]

**Supplementary tables**

**Table S1. List of primers used in this study.**

**A. List of primers concerning RTLV2.**

| Primer name | Sequence (5'-3') | Description |
| --- | --- | --- |
| RTLV2-5'F | CCAATCGTTAGTTTTACAGCAAAC | RT-PCR, fragment 1 of 4 |
| RTLV2-R1 | CTGTTGTTTGACGGAGAGCA |  |
| RTLV2-F2 | ACTTTTGATGTGCGAGCTGT | RT-PCR, fragment 2 of 4 |
| RTLV2-R2 | CGTAGGTCCAGTGTTCTTCAG |  |
| RTLV2-F3 | GGCATCTCTTCATCTCGTTGG | RT-PCR, fragment 3 of 4 |
| RTLV2-R3 | AGATCTCTTCAGCAGGTTTGTG |  |
| RTLV2-F4 | TGGAGCGGATCACTGATATAGG | RT-PCR, fragment 4 of 4 |
| RTLV2-3'R | GCCTAAGAATATAAAGGAGGAGAAG |  |
| RTLV2-R1 | CTGTTGTTTGACGGAGAGCA | Reverse transcription in 5' RACE |
| RTLV2-R1-2 | GCTCGCACATCAAAAGTCCA |  |
| rtlv2-5R-2r | ACCCACGACATCAAGAAGAGC | 5' RACE |
| rtlv2-5R-3r | CACTGTCTCGGTAGTTGGCA |  |
| rtlv2YJ-5R-4r | GATCCACATGGAGGAGATCATC |  |
| rtlv2YJ-5R-5r | ATCACACTCTATATAAGGCACACC |  |
| rtlv2-3R-2f | GCTCGTGGTAACACTGTGGA | 3' RACE, 1st trial |
| rtlv2-3R-4f | GAGTCATGCCCTATACAGAATCTG |  |
| rtlv2-3R-4.1f | GTCGGAGAGAGTTGCTGTAGTTG |  |
| rtlv2-3R-4.2f | CTTGCGTCATCAGAGTTTCTTAG |  |
| rtlv2-3R-5f | TCTGAACTGCAGTGGTACGG |  |
| rtlv2-3R-f1 | AGGGGTGCTGAGTTTCTGTC | 3' RACE, 2nd trial |
| rtlv2-3R-f2 | GGCAGGGACTCTCCACAATG |  |
| rtlv2-3R-f3 | GCGCTGTTGTGTTGACTTCT |  |
| rtlv2-3R-f4 | TTCTTGGTGCATTTCCCGGT |  |
| rtlv2-3R-f5 | GGTGGTTTTCCCGAGACCTT |  |
| 2270 2-2F | TAAATACTGGACTTTTGATGTGCG | RT-PCR detection, primer set 1 |
| 2271 2-2R | AAGTGCAGAAATATCTTCAGGCCA |  |
| 2261 3F | GAATCTATTGAAACAAGAGAGT | RT-PCR detection, primer set 2 |
| 2262 3R | AGGTAAGAGTCAAGGGTCCTATA |  |

**B. List of primers concerning RTLV1.**

| Primer name | Sequence (5'-3') | Description |
| --- | --- | --- |
| RTLV1-5'F | TATAGCTATCAAGAAATTCCAACTTA | RT-PCR, fragment 1 of 6 |
| RTLV1-R1 | TATCTGCTGTCCAACGATC |  |
| RTLV1-F2 | AGCTTGTGGTGAAATTCTGGT | RT-PCR, fragment 2 of 6 |
| RTLV1-R2 | CGACTCCCTTTCCTGAATCG |  |
| RTLV1-F3 | GAGCGGGATTTGTGGATTCA | RT-PCR, fragment 3 of 6 |
| RTLV1-R3 | CGGCTACATCTACAGGACATCT |  |
| RTLV1-F4 | ATGGGTGATCGTGCTCAG | RT-PCR, fragment 4 of 6 |
| RTLV1-R4 | CCCTCAGGTGGTTCAGAGAA |  |
| RTLV1-F5 | TCTGCACTTTGGTCAGTACTT | RT-PCR, fragment 5 of 6 |
| RTLV1-R5 | CCGAATCACACTGGACGAC |  |
| rtlv1-3R-4f | ACACCGTCTTGCGACTCTTT | RT-PCR, fragment 6 of 6 |
| rtlv1-3'R-2 | CTGACCCTGCACAGCATTAG |  |
| RTLV1-R1-2 | ACCAGAATTTCACCACAAGTTGT | Reverse transcription in 5' RACE |
| RTLV1-R1-3 | TAGCCTCTGTTGTTTGACGT |  |
| rtlv1-5R-1r | AATGAACCGAGACATCAGCCA | 5' RACE |
| rtlv1-5R-2r | ACCAGATGTGTCCCCTCCAA |  |
| rtlv1-5R-3r | GGTACCTCACGCACATTGAGA |  |
| rtlv1-5R-4r | CGTTGAGCAATGGCAAACAGA |  |
| rtlv1-3R-1f | ACATGCCATATCTTGAGAATGAGAG | 3' RACE, 1st trial |
| rtlv1-3R-2f | GGTGAGTGTTGCTGAGAGGT |  |
| rtlv1-3R-3f | GCAGCCCTCTATGCAAAAGC |  |
| rtlv1-3R-4f | ACACCGTCTTGCGACTCTTT |  |
| rtlv1-3R-5f | TCCGTCGTCCAGTGTGATTC |  |
| rtlv1-3R-f1 | TCAGACAACTGCCGATGGAA | 3' RACE, 2nd trial |
| rtlv1-3R-f2 | ACCTCGAAAGTGGTGCATCG |  |
| rtlv1-3R-f3 | AGAAAGTGCTGGCTACCGTC |  |
| rtlv1-3R-f4 | ACTTTTCTTGCGCTGTGGTC |  |
| rtlv1-3R-f5 | AAGAGTTGTGTGGCGGTTGA |  |
| 2247 TMV1F | AGAAATTCCAACTTACTAGTTATACA | RT-PCR detection, primer set 1 |
| 2248 TMV1R | ACCCTGTGATTCAATTTAGAGTGAGGA |  |
| 2249 TMV2F | GCCAACTTCTACTCAATAGAACGGGT | RT-PCR detection, primer set 2 |
| 2250 TMV2R | AGGAGAGTCAAAAGATTCATGAATCC |  |

**Table S2. Swissprot annotation of viral unigenes assembled in RNA-seq analysis of rubber tree bark samples.** RTV1, Rubber tree virus 1, a member of genus *Capillovirus*, family *Betaflexiviridae*.

| Unigene_id | Gene Length (nt) | Readcount* | Swissprot accession | Swissprot Description | E-value | Virus species |
| --- | --- | --- | --- | --- | --- | --- |
| Cluster-5029.6529 | 6364 | 1662.73 | P54891 | RNA-directed RNA polymerase, OS=Apple chlorotic leaf spot virus | 7.20E-138 | RTV1 |
| Cluster-5029.7651 | 5885 | 1726.84 | P27738 | RNA-directed RNA polymerase, OS=Apple chlorotic leaf spot virus | 2.50E-140 | RTV1 |
| Cluster-5029.36191 | 5099 | 1068.97 | P54891 | RNA-directed RNA polymerase, OS=Apple chlorotic leaf spot virus | 8.40E-137 | RTV1 |
| Cluster-5029.4122 | 3493 | 861 | Q91QZ3 | RNA replication polyprotein, OS=Citrus leaf blotch virus | 7.90E-46 | RTV1 |
| Cluster-5029.33260 | 1685 | 363 | Q64962 | RNA replication polyprotein, OS=Apple stem pitting virus | 9.50E-122 | RTV1 |
| Cluster-5029.6152 | 1432 | 278.34 | P36309 | Genome polyprotein, OS=Apple stem grooving virus | 5.20E-20 | RTV1 |
| Cluster-5029.6151 | 1432 | 396.66 | P36309 | Genome polyprotein, OS=Apple stem grooving virus | 5.20E-20 | RTV1 |
| Cluster-5029.3710 | 1109 | 148.19 | P54892 | Putative movement protein, OS=Apple chlorotic leaf spot virus | 2.50E-14 | RTV1 |
| Cluster-5029.33593 | 10352 | 587.56 | O93058 | Replicase large subunit, OS=Tobacco mosaic virus | 4.50E-97 | RTLV1 |
| Cluster-5029.36267 | 10340 | 151.1 | O93058 | Replicase large subunit, OS=Tobacco mosaic virus | 4.50E-97 | RTLV1 |
| Cluster-5029.34672 | 9361 | 4548.78 | O93058 | Replicase large subunit, OS=Tobacco mosaic virus | 4.10E-97 | RTLV1 |
| Cluster-5029.4006 | 9349 | 1119.45 | O93058 | Replicase large subunit, OS=Tobacco mosaic virus | 4.10E-97 | RTLV1 |
| Cluster-5029.5429 | 6190 | 1580.12 | O93058 | Replicase large subunit, OS=Tobacco mosaic virus | 2.70E-97 | RTLV1 |
| Cluster-5029.34673 | 6025 | 2439.13 | O93058 | Replicase large subunit, OS=Tobacco mosaic virus | 2.60E-97 | RTLV1 |
| Cluster-5029.37132 | 8796 | 1073.38 | P89676 | Replicase large subunit, OS=Tomato mosaic virus | 1.20E-95 | RTLV2 |
| Cluster-5029.4897 | 8344 | 4055 | P89676 | Replicase large subunit, OS=Tomato mosaic virus | 1.20E-95 | RTLV2 |
| Cluster-5029.4964 | 8224 | 1683.79 | P89676 | Replicase large subunit, OS=Tomato mosaic virus | 1.20E-95 | RTLV2 |
| Cluster-5029.4965 | 8211 | 1532.83 | P89676 | Replicase large subunit, OS=Tomato mosaic virus | 1.20E-95 | RTLV2 |

* Quantified by RSEM v1.2.15 (doi: 10.1186/1471-2105-12-323).

**Table S3. Similarity search of replicase-encoding ORF1 against NCBI NR database.** Hits were arranged by max score (accessed June 2023).

**A. Description of homologues found for RTLV1 ORF1.**

|  | Virus species | Classification | Host/origin | Protein description | aa length | Protein accession | Max Score | Query Cover | E-value | Identity |
| --- | --- | --- | --- | --- | --- | --- | --- | --- | --- | --- |
| 1 | Oxera neriifolia associated virus | unclassified | plant | polyprotein | 2487 | CAI5383846.1 | 676 | 71% | 0.0 | 37.89% |
| 2 | Hubei virga-like virus 11 | unclassified | invertebrate | hypothetical protein | 1065 | YP_009337242.1 | 452 | 37% | 2E-130 | 30.51% |
| 3 | Hubei virga-like virus 10 | unclassified | invertebrate | hypothetical protein | 3002 | APG77802.1 | 446 | 60% | 2E-121 | 31.70% |
| 4 | Plumeria mosaic virus | *Virgaviridae*; *Tobamovirus* | plant | large replicase | 1650 | WGL47827.1 | 427 | 48% | 7E-118 | 32.49% |
| 5 | Frangipani mosaic virus | *Virgaviridae*; *Tobamovirus* | plant | replicase large protein | 1650 | AEW67306.2 | 417 | 47% | 1E-114 | 33.09% |
| 6 | Atrato Virga-like virus 6 | unclassified | invertebrate | polyprotein | 2529 | QHA33758.1 | 405 | 53% | 3E-109 | 29.42% |
| 7 | Atrato Virga-like virus 7 | unclassified | invertebrate | polyprotein | 2529 | QHA33782.1 | 405 | 53% | 4E-109 | 29.42% |
| 8 | XiangYun hepe-virga-like virus 5 | unclassified | invertebrate | putative RNA-dependent RNA-polymerase | 2454 | UUG74081.1 | 404 | 52% | 6E-109 | 31.56% |
| 9 | Pedersore virga-like virus | unclassified | invertebrate | polyprotein | 1287 | UYL94373.1 | 392 | 32% | 2E-108 | 32.30% |
| 10 | Bactrocera dorsalis negev-like virus | unclassified | invertebrate | hypothetical protein 1 | 2171 | UPT53668.1 | 386 | 51% | 1E-103 | 30.95% |
| 11 | Yunnan virgavirus 3 | unclassified | cattle feces | putative 183 kDa protein | 1624 | QYW08488.1 | 385 | 47% | 3E-104 | 31.25% |
| 12 | Myzus persicae nege-like virus 1 | unclassified | invertebrate | replicase protein | 2576 | UTQ79656.1 | 382 | 57% | 5E-102 | 40.76% |
| 13 | Cactus tobamovirus 2 | unclassified | plant | RNA-dependent RNA polymerase | 1641 | UPI40893.1 | 381 | 42% | 4E-103 | 31.52% |
| 14 | Opuntia virus 2 | unclassified | plant | 187 kDa hypothetical polymerase | 1598 | YP_009553013.2 | 374 | 47% | 5E-101 | 31.78% |
| 15 | Passion fruit mosaic virus | *Virgaviridae*; *Tobamovirus* | plant | unnamed protein product | 1611 | YP_004465358.1 | 373 | 45% | 1E-100 | 31.35% |
| 16 | Clitoria yellow mottle virus | *Virgaviridae*; *Tobamovirus* | plant | unnamed protein product | 1633 | YP_004956727.1 | 369 | 54% | 2E-99 | 30.40% |
| 17 | Paprika mild mottle virus | *Virgaviridae*; *Tobamovirus* | plant | RdRp | 1616 | ANV28178.1 | 366 | 45% | 2E-98 | 30.56% |
| 18 | Sewage-associated tobamovirus | unclassified | sewage water | large replicase protein | 1625 | BDW14813.1 | 365 | 47% | 3E-98 | 31.71% |
| 19 | Cactus tobamovirus 1 | unclassified | plant | RNA-dependent RNA polymerase | 1638 | UPI40897.1 | 364 | 42% | 8E-98 | 31.53% |
| 20 | Hoya necrotic spot virus | unclassified | plant | RNA replicase read-through component | 1624 | QOC69614.1 | 364 | 46% | 1E-97 | 30.02% |
| 21 | Guangxi virgavirus | unclassified | cattle feces | putative replicase | 1621 | QYW08462.1 | 363 | 42% | 1E-97 | 31.53% |
| 22 | Tomato mottle mosaic virus | *Virgaviridae*; *Tobamovirus* | plant | RNA-dependent RNA polymerase | 1616 | UOF93479.1 | 363 | 45% | 2E-97 | 32.16% |
| 23 | Tomato mosaic virus | *Virgaviridae*; *Tobamovirus* | plant | 183 kDa protein | 1616 | UIX55990.1 | 362 | 45% | 3E-97 | 31.64% |
| 24 | Cactus mild mottle virus | *Virgaviridae*; *Tobamovirus* | plant | replicase | 1649 | YP_002455905.1 | 362 | 42% | 4E-97 | 31.54% |
| 25 | Rehmannia mosaic virus | *Virgaviridae*; *Tobamovirus* | plant | putative replicase | 1616 | AVK94647.1 | 360 | 45% | 1E-96 | 31.17% |

**B. Description of homologues found for RTLV2 ORF1.**

|  | Virus species | Classification | Host/origin | Protein description | aa length | Protein accession | Max Score | Query Cover | E-value | Identity |
| --- | --- | --- | --- | --- | --- | --- | --- | --- | --- | --- |
| 1 | Oxera neriifolia associated virus | unclassified | plant | polyprotein | 2487 | CAI5383846.1 | 687 | 73% | 0.0 | 38.66% |
| 2 | Adelphocoris suturalis virus | unclassified | invertebrate | ORF1 | 2592 | YP_009336476.1 | 439 | 49% | 1E-119 | 33.41% |
| 3 | Atrato Virga-like virus 7 | unclassified | invertebrate | polyprotein | 2536 | QHA33778.1 | 425 | 56% | 2E-115 | 30.15% |
| 4 | Atrato Virga-like virus 6 | unclassified | invertebrate | polyprotein | 2421 | QHA33762.1 | 422 | 56% | 2E-114 | 30.06% |
| 5 | Plumeria mosaic virus | *Virgaviridae*; *Tobamovirus* | plant | large replicase | 1650 | WGL47827.1 | 395 | 53% | 2E-107 | 30.01% |
| 6 | Frangipani mosaic virus | *Virgaviridae*; *Tobamovirus* | plant | large replicase | 1650 | WGL47831.1 | 393 | 55% | 5E-107 | 29.17% |
| 7 | Hubei sediment virgavirus 1 | unclassified | lake sediment | putative polymerase | 1676 | QYW08476.1 | 382 | 44% | 3E-103 | 31.64% |
| 8 | Hubei sediment virgavirus 2 | unclassified | lake sediment | putative polymerase | 1675 | QYW08480.1 | 379 | 44% | 3E-102 | 31.71% |
| 9 | Eriocheir sinensis tobamo-like virus | unclassified | invertebrate | polyprotein | 1675 | WCF59102.1 | 378 | 44% | 5E-102 | 31.71% |
| 10 | Myzus persicae nege-like virus 1 | unclassified | invertebrate | replicase protein | 2576 | UTQ79656.1 | 373 | 60% | 2E-99 | 41.20% |
| 11 | Tomato mosaic virus | *Virgaviridae*; *Tobamovirus* | plant | 183 kDa protein | 1616 | UIX55990.1 | 360 | 45% | 1E-96 | 32.80% |
| 12 | Cactus tobamovirus 2 | unclassified | plant | RNA-dependent RNA polymerase | 1641 | UPI40905.1 | 360 | 43% | 3E-96 | 31.41% |
| 13 | Rattail cactus necrosis-associated virus | *Virgaviridae*; *Tobamovirus* | plant | RdRp protein | 1642 | ATU47241.1 | 359 | 43% | 3E-96 | 32.79% |
| 14 | Plantago tobamovirus 1 | unclassified | plant | replicase polyprotein | 1601 | UVK78386.1 | 358 | 47% | 6E-96 | 31.59% |
| 15 | Tomato mottle mosaic virus | *Virgaviridae*; *Tobamovirus* | plant | RNA-dependent RNA polymerase | 1616 | UOF93479.1 | 358 | 45% | 6E-96 | 32.55% |
| 16 | Clitoria yellow mottle virus | *Virgaviridae*; *Tobamovirus* | plant | unnamed protein product | 1633 | YP_004956727.1 | 357 | 47% | 1E-95 | 33.17% |
| 17 | Sewage-associated tobamovirus | unclassified | sewage water | large replicase protein | 1625 | BDW14813.1 | 357 | 46% | 1E-95 | 32.62% |
| 18 | Cactus tobamovirus 1 | unclassified | plant | RNA-dependent RNA polymerase | 1638 | UPI40897.1 | 357 | 43% | 2E-95 | 32.31% |
| 19 | Turnip vein-clearing virus | *Virgaviridae*; *Tobamovirus* | plant | RNA replicase | 1601 | AEU04723.1 | 356 | 47% | 2E-95 | 32.15% |
| 20 | Ribgrass mosaic virus | *Virgaviridae*; *Tobamovirus* | plant | replicase readthrough component | 908 | QIJ70152.1 | 344 | 30% | 2E-95 | 31.80% |

**Table S4. Similarity search of CP-encoding ORF4 against NCBI NR database.** Hits were arranged by max score (accessed June 2023).

**A. Description of homologues found for RTLV1 ORF4.**

|  | Virus species | Classification | Host/origin | Protein description | aa length | Protein accession | Max Score | Query Cover | E-value | Identity |
| --- | --- | --- | --- | --- | --- | --- | --- | --- | --- | --- |
| 1 | Culex pipiens-associated Tunisia virus | unclassified | invertebrate | capsid | 160 | YP_009553258.1 | 85.5 | 92% | 3E-17 | 31.68% |
| 2 | Atrato Virga-like virus 5 | unclassified | invertebrate | putative capsid protein | 159 | QHA33753.1 | 83.2 | 93% | 2E-16 | 32.12% |
| 3 | Adelphocoris suturalis virus | unclassified | invertebrate | ORF5 | 159 | YP_009336480.1 | 68.6 | 93% | 1E-10 | 26.22% |
| 4 | Bemisia tabaci bromo-like virus 1 | unclassified | invertebrate | coat protein | 144 | QWC36508.1 | 66.2 | 91% | 5E-10 | 27.44% |
| 5 | Diabrotica undecimpunctata virus 2 | unclassified | invertebrate | putative structural protein | 219 | QIT20100.1 | 55.8 | 91% | 0.00001 | 26.25% |
| 6 | Xiangshan martelli-like virus 3 | unclassified | invertebrate | hypothetical protein | 235 | UDL14012.1 | 53.9 | 83% | 0.00007 | 22.60% |
| 7 | Beihai charybdis crab virus 1 | unclassified | invertebrate | putative capsid protein | 154 | YP_009333243.1 | 53.5 | 94% | 0.00004 | 26.79% |
| 8 | Atrato Virga-like virus 3 | unclassified | invertebrate | putative coat protein | 196 | QHA33743.1 | 53.5 | 80% | 0.00006 | 25.00% |
| 9 | Atrato Virga-like virus 1 | unclassified | invertebrate | putative coat protein | 207 | QHA33735.1 | 53.5 | 93% | 0.00006 | 25.60% |
| 10 | Hubei virga-like virus 1 | unclassified | invertebrate | hypothetical protein | 246 | YP_009337424.1 | 52 | 91% | 0.0003 | 22.01% |
| 11 | Alexandroupolis virga-like virus | unclassified | invertebrate | putative coat protein | 199 | QRD99903.1 | 51.2 | 74% | 0.0004 | 27.91% |
| 12 | Atrato Virga-like virus 2 | unclassified | invertebrate | putative coat protein | 180 | QHA33737.1 | 51.2 | 81% | 0.0004 | 27.59% |
| 13 | Hubei virga-like virus 2 | unclassified | invertebrate | putative coat protein | 205 | UUG74048.1 | 51.2 | 76% | 0.0005 | 27.82% |
| 14 | Plant associated tobamo-like virus 1 | unclassified | plant | 29 kDa putative coat protein | 253 | UTQ50505.1 | 51.2 | 85% | 0.0006 | 28.86% |
| 15 | Pedersore virga-like virus | unclassified | invertebrate | polyprotein | 323 | UYL94366.1 | 48.9 | 91% | 0.005 | 26.25% |
| 16 | Bemisia tabaci virga-like virus 2 | unclassified | invertebrate | putative coat protein | 169 | QWC36454.1 | 47.4 | 94% | 0.007 | 27.54% |

**B. Description of homologues found for RTLV2 ORF4.**

|  | Virus species | Classification | Host/origin | Protein description | aa length | Protein accession | Max Score | Query Cover | E-value | Identity |
| --- | --- | --- | --- | --- | --- | --- | --- | --- | --- | --- |
| 1 | Culex pipiens-associated Tunisia virus | unclassified | invertebrate | capsid protein | 160 | QRW42066.1 | 92.4 | 83% | 1E-19 | 33.54% |
| 2 | Atrato Virga-like virus 5 | unclassified | invertebrate | putative capsid protein | 159 | QHA33753.1 | 85.5 | 84% | 4E-17 | 31.48% |
| 3 | Hubei virga-like virus 1 | unclassified | invertebrate | hypothetical protein | 246 | YP_009337424.1 | 63.5 | 82% | 3E-08 | 25.16% |
| 4 | Myzus persicae nege-like virus 1 | unclassified | invertebrate | hypothetical protein 3 | 169 | UTQ79659.1 | 61.2 | 83% | 8E-08 | 25.31% |
| 5 | Xiangshan martelli-like virus 3 | unclassified | invertebrate | hypothetical protein | 235 | UDL14012.1 | 53.9 | 76% | 0.0001 | 25.34% |
| 6 | Goji berry chlorosis virus | unclassified | plant | coat protein | 156 | AYO99570.1 | 52.4 | 78% | 0.0001 | 28.76% |
| 7 | Megastigmus ssRNA virus | unclassified | invertebrate | hypothetical protein | 437 | QDZ71187.1 | 52 | 82% | 0.0007 | 20.25% |
| 8 | Beihai charybdis crab virus 1 | unclassified | invertebrate | putative capsid protein | 154 | YP_009333243.1 | 50.1 | 48% | 0.0007 | 27.55% |
| 9 | Atrato Virga-like virus 2 | unclassified | invertebrate | putative coat protein | 180 | QHA33737.1 | 47.4 | 82% | 0.011 | 25.31% |
| 10 | Atrato Virga-like virus 3 | unclassified | invertebrate | putative coat protein | 196 | QHA33743.1 | 46.6 | 76% | 0.025 | 23.97% |
