# Supplementary material for: Identification and characterization of two closely related virga-like viruses latently infecting rubber trees (Hevea brasiliensis)
Source: Front Microbiol. 2023 Dec 14;14:1286369. doi: 10.3389/fmicb.2023.1286369 (PMC10752949; doi:10.3389/fmicb.2023.1286369)
Supplement: Supplementary file 2 [file Data_Sheet_1.docx]

**Supplementary Figures**


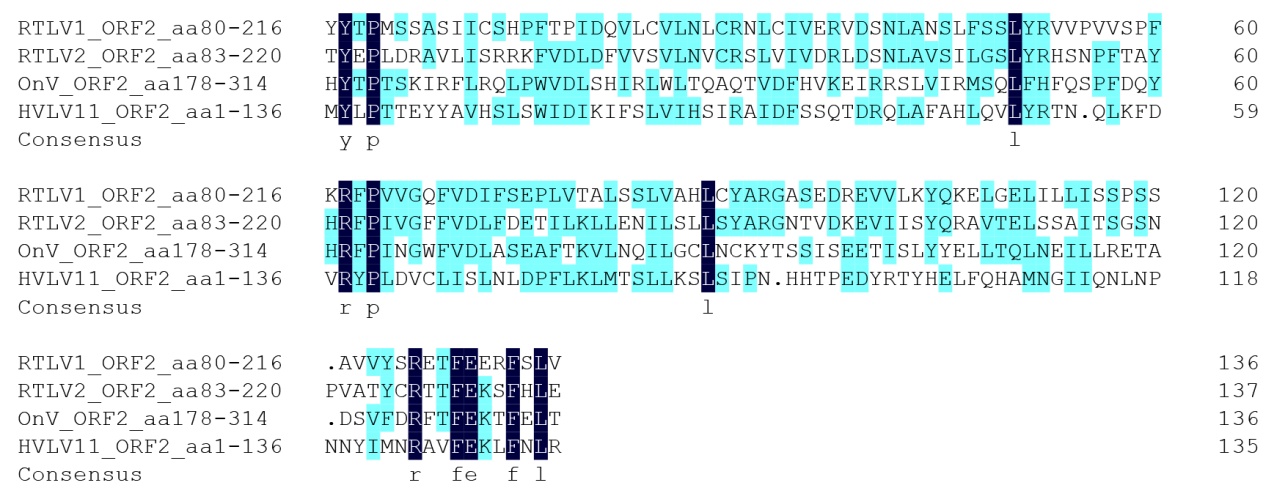


**Supplementary Figure 1. Multiple alignments of ORF2 of RTLV1, RTLV2, OnV and HVLV11.** Identical residues were highlighted by dark blue and residues with homology level ≥50% were highlighted by light blue (calculated by DNAMAN). GenBank accession: OnV, Oxera neriifolia associated virus, OX380366 (ORF2, CAI5383847); HVLV11, Hubei virga-like virus 11, MF348194 (ORF2, AVK59470).

**
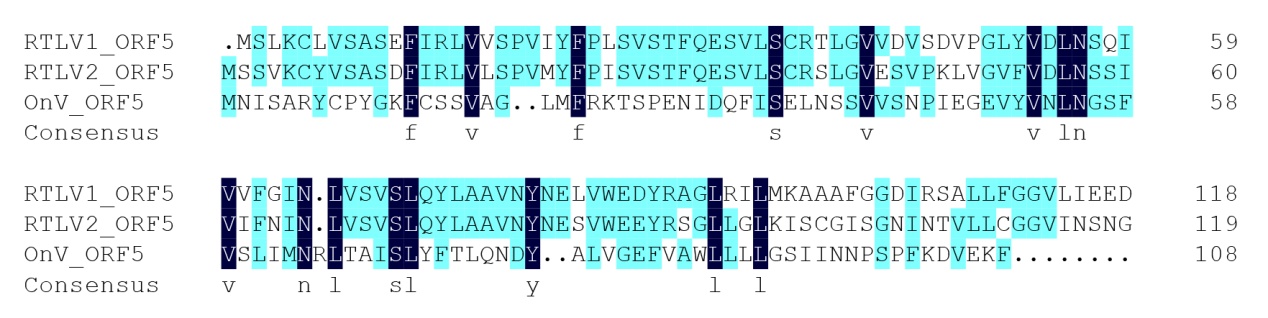
**

**Supplementary Figure 2. Multiple alignments of ORF5 of RTLV1, RTLV2 and OnV.** Identical residues were highlighted by dark blue and residues with homology level ≥50% were highlighted by light blue (calculated by DNAMAN). GenBank accession: OnV, Oxera neriifolia associated virus, OX380366 (ORF5, CAI5383850).
